# End-of-life in cancer patients: Medicolegal implications and ethical challenges in Europe

**DOI:** 10.1515/med-2025-1218

**Published:** 2025-06-10

**Authors:** Susanna Marinelli, Nicola Di Fazio, Miriam Ottaviani, Gianpietro Volonnino, Simona Zaami, Lina De Paola

**Affiliations:** School of Law, Polytechnic University of Marche, Ancona, 60121, Italy; Department of Life Sciences, Health and Health Professions, Link Campus University, Rome, Italy; Department of Anatomical, Histological, Forensic and Orthopedic Sciences, Sapienza University of Rome, Rome, Italy

**Keywords:** oncology, end-of-life, ethical/legal implication, cancer

## Abstract

**Introduction:**

This study explores the complexities and distinctive traits of end-of-life (EOL) care and assisted suicide in cancer patients across Europe, and the challenges they entail. It analyzes various countries in the Southern, Central, and Northern regions. Legal, ethical, and cultural dimensions of euthanasia are examined. Differences in practices across Europe are highlighted.

**Materials and methods:**

The aim of this study is to provide an overview of EOL care policies in Europe by delving into the legislative/policy-making approaches of three selected nations, and implications thereof.

**Results:**

Disparities between regions are identified, to figure out margins for improvement. This includes advocating for a balanced approach that both upholds legal frameworks and respects patient autonomy. By doing so, the ultimate objective is to foster a culture of ethical and empathetic EOL care for cancer patients throughout Europe, ensuring that their needs and preferences are prioritized till the end. Advocacy for a balanced approach is recommended.

**Conclusion:**

Ultimately, the findings herein presented point to the need for a collaborative effort among policymakers, healthcare providers, and communities to build a more holistic approach to end-of-life care that harmonizes legal regulations with the ethical imperative of respecting individual choice in an environment marked by sensitivity and compassion.

## 1 Introduction

Cancer rates in Europe are unfortunately still rather considerable: each year, more than 3 million new cancer cases are diagnosed in Europe, affecting approximately 1.6 million men and 1.4 million women. The cancer diagnosis is always devastating: treatments are demanding, the impact on the patient's life can be significant, and the prognosis is not always favorable. According to the International Agency for Research on Cancer (IARC), in Europe in 2022, nearly 4.5 million new cancer cases were reported, representing 22% of the global total, including almost 2.5 million in men and just over 2 million in women [1].

The report “The Numbers of Cancer 2023” estimates that in 2023, cancer cases increased by over 18,000, reaching approximately 395,000 cases (208,000 in men and 187,000 in women) [2]. In the United Kingdom, according to IARC, the three most diagnosed types of cancer were, in descending order, breast, prostate, and lung cancer, with 181,807 deaths. In France, the leading cancers were breast, prostate, and colorectal, with 190,612 deaths. In Italy, the most common cancers were breast, colorectal, and lung, with 193,706 cancer-related deaths [1].

However, thanks to advancements in scientific research over the last 30 years, a cancer diagnosis no longer equates to a death sentence. If diagnosed early, cancer is more likely to be successfully treated. Within the European Union, around 40% of cancers are estimated to be potentially preventable, and there are more than 12 million cancer survivors [3,4].

However, in cases where the diagnosis unfortunately carries a poor prognosis, individuals may choose to end their lives autonomously, in a way that may be more “acceptable” to them and their families [5,6]. Additionally, they have the right to access palliative treatments that can improve their quality of life in their final moments. Indeed, in every country, there are laws that allow assisted suicide or access to palliative care. The end-of-life (EOL) phase is, for cancer patients, a period marked by significant changes that interfere with their daily lives. The considerations surrounding this difficult period are numerous and span legal, ethical, social, and psychological dimensions [7,8]. In Europe, varying legal frameworks, cultural attitudes, and healthcare systems contribute to the diverse ways in which EOL issues are addressed. This complexity is further compounded by the evolving role of palliative care, assisted suicide, and the ethical dilemmas surrounding life-sustaining treatments vs patient autonomy.

In recent years, there has been an effort to make this phase more balanced, especially concerning the rights of individuals with cancer [9]. The issue of transitioning from oncological care to palliative treatments while respecting patients' wishes to die according to their own preferences, as well as their psychological needs and those of their families or caregivers, has become central. The attention from the relevant authorities has grown increasingly broader. Furthermore, from a medico-legal perspective, the responsibility of healthcare professionals in cases involving end-of- life decisions and informed consent is also a focus. Preventing cases of medical negligence in these situations is essential to protect both doctors and patients, as well as their loved ones. The aim of this study is to explore the medico-legal implications and ethical challenges associated with EOL care for cancer patients in Europe, focusing on current practices, legal frameworks, and ethical debates. The research examines and compares the current national regulations in selected European countries, geographically categorized into Northern, Central, and Southern Europe, and their ethical impact in cases involving cancer patients facing end-of-life decisions.

## 2 Materials and methods

**Figure 1:**
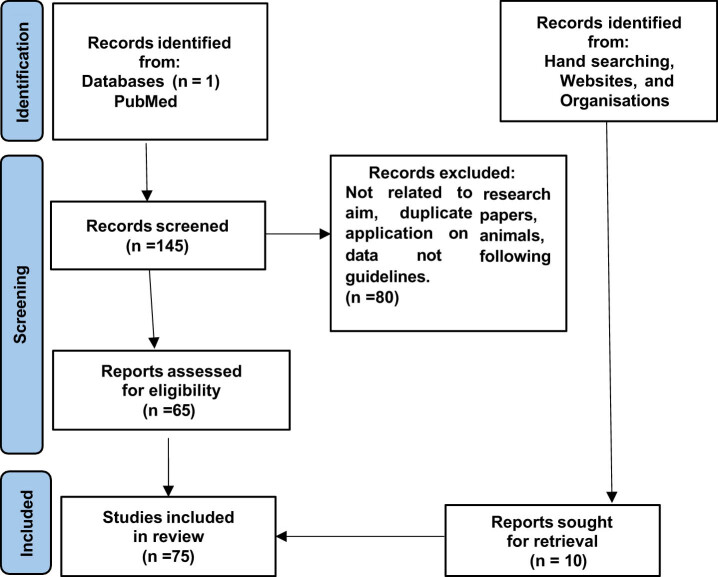
Prisma flow chart.

The study analyzed articles available on PubMed from the past 5 years. Using the search terms “end of life,” “cancer patients,” “European,” and “palliative care,” 145 results were found. Articles identified through databases were screened by title and abstract. The full texts of potentially relevant articles were then analyzed and reviewed to assess their eligibility. Only articles written in English and addressing the topic of EOL care were included. Duplicates and results unrelated to the subject matter were excluded. We included all types of papers, from case reports to reviews, with no exclusion criteria, to ensure our work was as comprehensive as possible. Ultimately, as summarized in Figure 1, 65 articles were deemed suitable for inclusion in this study. Additional data were incorporated after manually searching websites and organizational documents to better understand the focus of the study. National and international legal, judicial, and regulatory sources were also considered, limited to European countries.

To enhance the study, supplementary data were collected through manual searches of websites and organizational documents, enabling a deeper understanding of the context and integrating information not readily accessible through traditional methods. Additionally, legal, judicial, and regulatory sources were examined at both national and international levels to situate the study within the relevant legal and regulatory framework, identify pertinent constraints or opportunities, and compare national practices with international standards. We consulted the websites of the WHO, the governments of the selected countries, particularly the Ministry of Health's website. We also reviewed the websites of international palliative care organizations and the official records of UN assemblies. This mixed-method approach, combining systematic research with manual analysis, contributed to a more comprehensive and nuanced understanding of the research topic.

The study aims to analyze the current state of EOL care in oncological patients, evaluating ethical and medico-legal implications in different European countries.

## 3 Results

The advances in medicine and the extension of life expectancy have raised issues that were previously hidden by a general reluctance to discuss suicide [10]. This gradual openness has led to an increasing number of countries in Europe and around the world acknowledging the possibility for citizens to access methods of ending their lives. In recent decades, medical practice has largely moved beyond the paternalistic model, recognizing the autonomy of patients, even in matters related to the EOL [11]. The two main forms of EOL care, euthanasia, and physician-assisted suicide, have been introduced into the healthcare system in various countries [12,13].

In most legislations, the applicant must have a conscious, free, and clearly stated will, which is verified, to be admitted to euthanasia/physician-assisted suicide (E/PAS) practices. Furthermore, external pressures or personal advantages for others are prohibited. As reported by the Italian National Bioethics Committee, the legal admissibility of EOL practices has been extended in several countries not only to terminal physical diseases but also to physical and mental medical conditions that cause prolonged and unbearable suffering, for which reasonable therapeutic alternatives are absent [14,15].

The EOL situation for cancer patients in the United Kingdom (Northern Europe), France (Central Europe), and Italy (Southern Europe) presents some differences, but also several common points, especially when it comes to the approach of palliative care and patient rights, as shown in Table 1.

**Table 1:** EOL situation for cancer patients in the United Kingdom (Northern Europe), France (Central Europe), and Italy (Southern Europe)

Country	Criteria	Euthanasia	Assisted suicide	Palliative sedation	Advance healthcare directives (AHDs)
United Kingdom	(Under discussion)	Prohibited	Prohibited (debate ongoing). Refusal of treatment allowed	Permitted: Advanced palliative care system, integrated into the national health system (NHS)	Recognized: “Advanced Decision” (Living Will) to refuse specific treatments in advance
	Terminal illness (<6 months survival)				
France	Refractory suffering to pharmacolog ical treatments	Illegal	Illegal, but “deep and continuous sedation” allowed in certain cases sanctioned by law	Permitted: The Léonetti Law (2005, updated 2016) guarantees access to palliative care, deep sedation for terminal patients, introduces a “person of trust,” and reshapes advance directives so they are binding	Binding: “Directives Anticipées”
	Terminal illness				
Italy	Patient fully capable of understanding and willing	Prohibited	Illegal, but not punishable in specific cases (Constitutional Court, 2019)	Permitted: Law 38/2010 guarantees access to palliative care and pain therapy	Binding: Advance Health Directives (AHD) Law 219/2017, in effect since 2018
	Irreversible condition causing intolerable suffering (physical or psychological)				
	Vital dependence on machines or treatments				

While each country has its own healthcare system and regulations, they share a general commitment to providing compassionate care for those in the final stages of life, ensuring that patients have access to the support and services they need. However, the implementation and accessibility of such care can vary, influenced by cultural attitudes, political decisions, and healthcare infrastructure in each country [6,16,17].

### 3.1 United Kingdom

In the United Kingdom, assistance or encouragement of suicide is illegal, as established by the Suicide Act of 1961. Specifically, Section 1 decriminalizes suicide for those who attempt or carry it out, while Section 2 states that anyone who encourages or assists another person in committing or attempting suicide will be punished with imprisonment for up to 14 years. Therefore, at present, both euthanasia and assisted suicide are illegal in the UK [6,18]. The law reflects a clear stance on the issue, emphasizing the criminality of aiding or promoting suicide, while also decriminalizing the act of suicide itself for the individual. This legal framework is part of the broader approach to EOL issues, where other forms of medical intervention, such as palliative care, are promoted instead [19].

Perhaps, however, this historical period may mark a turning point for the country, potentially leading to the legalization of assisted suicide. Indeed, there are numerous movements and organizations advocating for this right, such as the charity “Dignity in Dying,” one of whose prominent figures is Esther Rantzen, a journalist, presenter, and well-known television personality, currently suffering from stage-four lung cancer. In an interview, Rantzen shared a poignant reflection: she realized that she envied the peaceful, painless, and compassionate death (“that merciful end”) that was afforded to her dog, a death she knows will be denied to her. She questions why, despite our love for animals, we treat them with far more dignity in their final moments than we do people [20,21].

Esther has already contacted the Swiss clinic *Dignitas* [22] because she knows that, given the time she has left, she will likely not live long enough to see any change in her country's laws. However, she has decided to continue fighting for the right of those who will come after her, especially since she understands that not everyone can afford the final trip to Switzerland, as she has been able to do. For this reason, together with the organization *Dignity in Dying*, she launched a petition that gathered 100,000 signatures in less than a month [23]. This initiative has undoubtedly sparked an important parliamentary debate, which culminated on October 16, 2024, with the presentation of the EOL bill by Labor MP Kim Leadbeater. However, this is not the first attempt to change the law in the United Kingdom: in 2015, the so-called “Marris Bill” was proposed and discussed in Parliament, while in 2021, it was followed by the “Meacher’s Bill” and, a few months later, Amendment 170 in relation to the “Health and Social Care Bill,” which addresses the reform of the British National Health Service [24].

All these proposed bills have failed to pass in Parliament, with the main argument put forth by opponents being the concern that such legislation would undermine the protection of vulnerable individuals and those with disabilities. In this regard, 1,700 British doctors expressed their stance in a letter to the UK Health Secretary, stating that they would refuse to perform assisted suicide procedures on patients, even if the bill were to be approved. According to them, “the cruel irony of this path is that the legislation, introduced with the good intention of enhancing (the right to) patient choice, would actually diminish the choices of the most vulnerable” [25], who might perceive themselves as a burden or a financial cost to their loved ones and feel pressured – either by this perception or by their families – to consider assisted suicide, a thought they might never have entertained otherwise.

In any case, the so-called “Meacher's Bill” proposed stringent requirements for accessing medically assisted suicide. These requirements essentially included being an adult, of sound mind and judgment, and “terminally ill,” meaning suffering from a progressive and irreversible illness in the terminal stage, with a life expectancy of less than 6 months [26].

A significant political endorsement for passing a bill on assisted suicide came from the current British Prime Minister and leader of the Labor Party, Keir Starmer, who had expressed support for the issue during his election campaign. He had already backed the assisted suicide bill in 2015 and recently declared that he was “very pleased” to be able to keep the promise he made to Esther Rantzen. The vote took place on November 29, 2024, and the British Parliament gave its initial approval to the bill, which will make assisted suicide legal in the UK for terminally ill adults with only 6 months to live, who can self-administer the fatal medication. Parliament approved the assisted dying bill by a 330–275 vote, signaling their approval in principle for the bill, which will undergo further scrutiny before it goes to a final vote [27].

With regard to the limitations of access to the palliative care system in the United Kingdom, as in France, the main obstacles are found in the variability of access in different areas of the country, due to the decentralized funding of the NHS, with approximately 40% of districts lacking sufficient palliative care provision, economic fluctuations also make services vulnerable, as hospices rely heavily on charitable donations, and the late availability of palliative care services means that many patients are only referred there if they have cancer and when it is very late, with more than 50% accessing in the last week of life [28].

### 3.2 France

The French legislation on EOL care has been recently amended with the adoption by Parliament of Law No. 2016-87 of February 2, 2016, which establishes new rights for patients and terminally ill individuals. This legislative development extends the provisions of the Léonetti Law of April 20, 2005, concerning patients’ rights at the EOL, and was enacted after several years of reflection marked by public and parliamentary debates [29]. As regards palliative care, France guarantees access to it for terminally ill patients with serious diseases in order to avoid patient suffering. Such palliative care is integrated into the NHS and is available both in hospitals and at home [30]. The successful implementation of this new law depends on the execution of the four areas of the Palliative and EOL Care Plan (CNAMTS), which are patient education to enable autonomous decision-making, professional training and research on the topic, home care, and guaranteed access to palliative care with the reduction in social inequalities. As for euthanasia and assisted suicide, these practices are still considered illegal. However, the law permits “sèdation profonde et continue” (deep and continuous sedation) until death for patients suffering from refractory pain and terminal diseases. This practice is allowed as it is believed to alleviate the suffering of individuals afflicted by incurable conditions without actually accelerating the process of dying [31].

French legislation also established the legal status of the *personne de confiance* (trusted person) in 2002, which refers to the decision-making substitute or healthcare proxy. The legal status of this individual was formalized in 2005 and is further strengthened in the current law. The *personne de confiance* expresses the patient's wishes, and the declarations made by this person take precedence over those of others. The attending physician is required to ensure that patients are informed about the possibility of designating a *personne de confiance* through a written document co-signed by the designated individual [32].

In France, patients can create “Directives Anticipées” (advance directives), a document that allows them to express their wishes regarding future medical treatments they may undergo, in a binding manner, though not entirely enforceable. The physician is required to respect the directives, except in three situations: if there is an emergency that endangers the patient's life, if the directives are manifestly inappropriate, or if they are incompatible with the patient's medical condition. The new law has expanded the scope of advance directives and extended their validity period. The goal is for advance directives to be used as a tool for dialogue, helping individuals with terminal illnesses anticipate the end of their life and express their personal decisions as accurately as possible [33].

The main limitations regarding access to the palliative care system in France, as in other countries, relate to geographical disparities (only about 60% of palliative care units are located in public hospitals, limiting accessibility in some areas), unequal access based on diagnosis (only 15–20% of people with non-cancer illnesses receive palliative care before death), and financial and structural constraints (funding is inconsistent, with heavy reliance on hospital budgets) [34,35].

In summary, French legislation provides an advanced legal framework for the management of end- of-life care, with a strong focus on patient autonomy. Although euthanasia and assisted suicide remain illegal, deep and continuous sedation is permitted to allay the suffering of terminal patients. The system aims to ensure respect for patients’ wishes while promoting open dialogue and support in EOL decisions.

### 3.3 Italy

The issue of EOL care is the subject of intense debate in Italy, with numerous ethical and political considerations. Some advocate for the legalization of euthanasia, while others believe it is essential to ensure access to palliative care and respect the patient's wishes through AHD [36,37,38,39]. The first innovations on this topic in Italy were introduced by Law No. 38 of 2010, which guarantees the right to the best possible treatment of suffering. This law, which also covers pain therapy, was one of the first in Europe to guarantee palliative care support for the patient and their family, including care in hospitals, at home, and in hospices. The law establishes that in Italy, palliative care, as an integrated part of healthcare, must be accessible to all citizens. It also stipulates that healthcare workers be trained in palliative care nationwide and that a fund be created to promote awareness of this issue at the local level through targeted informational campaigns. Furthermore, the decree emphasizes the importance of pain management as well as the periodic assessment of the quality of palliative care provided [40].

It is important to highlight that this law represents the culmination of a process already initiated with Law No. 39/1999, which established the start of the national program for home-based palliative care as well as the implementation of hospices, carried out through a series of agreements in the State-Region Conference [41].

The advancements of Law No. 38 of 2010 were further strengthened by the subsequent Law No. 219/2017, which enshrines the right to patient self-determination. This law reinforces the concept that, through informed consent, palliative care must be included within the patient’s therapeutic plan, even in the event of refusal or withdrawal of treatment by the patient. It also establishes the necessity for a shared care planning approach for the patient, including in the context of palliative treatment, as well as the need for adequate training of healthcare workers [39].

However, in Italy, there is significant territorial heterogeneity in the application of the law, partly due to the presence of different regional healthcare systems that enjoy considerable autonomy in relation to national government directives.

Regarding medically assisted suicide and euthanasia, it can be stated that active euthanasia, i.e., the act of directly causing a person’s death, is illegal in Italy and is considered homicide of the consenting person (Article 579 of the Italian Penal Code). However, the 2019 Constitutional Court ruling No. 242 [42] paved the way for medically assisted suicide by declaring that those who help a person die, under certain conditions, will not be punished. The main conditions are:

- The patient must be suffering from an incurable and irreversible disease.
- The patient must be kept alive by life-sustaining treatments.
- The disease must cause intolerable physical or psychological suffering.
- The patient must be fully capable of making decisions and understanding.
- The decision must be free and informed.

However, the provisions of this ruling, while innovative, raised some concerns regarding the interpretation of the concept of “life-sustaining treatment.”

Therefore, the subsequent Constitutional Court ruling No. 135/2024 [43], issued on July 18, 2024, clarified the meaning of this requirement, specifying that this expression does not refer exclusively to treatments that artificially keep the patient alive (such as mechanical ventilation), but also includes treatments that, while not essential for survival, support the patient's vital functions and allow them to live with greater autonomy and well-being. Examples of “life-sustaining treatments” are therefore artificial nutrition, artificial hydration, dialysis, chemotherapy therapies *quoad vitam*, and pharmacological therapies for pain control and other symptoms.

This clarification is important because it expands the number of patients who may potentially access medically assisted suicide. In fact, even patients who are not “kept alive” by machines, but who rely on treatments to alleviate their suffering and improve their quality-of-life, may fall within the criteria established by the Constitutional Court [44,45].

The Constitutional Court has strongly reiterated the need for a law that more precisely regulates medically assisted suicide, defining the procedures and necessary requirements [46]. This law is crucial to ensure that the patient's decision is free and informed, that their rights are respected, and that the doctors assisting them are protected.

However, the absence of a national law led the Region of Tuscany to adopt, in 2025, the first legislation to regulate when and how assisted suicide can be accessed. In fact, the most recent developments led to the approval, on 11 February 2025, of the first regional law on assisted suicide in Tuscany. The regulation sets 20 days (within which the Ethics Committee has 7 days to express its opinion) the maximum time to establish whether the requirements for access to assisted suicide are met, as established by ruling no. 242/2019. Upon a positive outcome, within a further 10 days, the modalities by which assisted suicide will take place, such as the choice of medication. After these 30 days in total, the rule guarantees, within 7 days, and with the support of the regional health system, the implementation of the procedure. Nevertheless, this law is not currently in force as its implementation has been suspended.

Currently, a bill is under discussion to more specifically and adequately regulate medically assisted suicide, outlining the procedures and necessary requirements.

To better clarify the situation in individual states, the authors have highlighted some of the selected articles in a more easily accessible table (Table 2). This table organizes the articles by title, authors, publication year, and key focus, providing a clear and concise overview of the literature used to describe EOL care.

**Table 2:** Selected articles describing EOL situations

State	Title	Authors	Publication year	Key focus
UK [6]	Advance care planning in patients with advanced cancer: A 6-country, cluster-randomised clinical trial	Korfage IJ, Carreras G, Arnfeldt Christensen CM, Billekens P, Bramley L, Briggs L, et al.	2020	This study suggests that alternative approaches to support patient-centered EOL care in this population are needed
UK [18]	Suicide Act 1961. Statute Law Database	Available online: https://www.legislation.gov.uk/ukpga/Eliz2/9-10/60	1961	It decriminalized suicide in England and Wales but made it a criminal offense to assist or encourage suicide, highlighting the importance of psychological support for those at risk
UK [19]	10 Years of End-of-Life Criteria in the United Kingdom	Bovenberg J, Penton H, Buyukkaramikli N	2021	New guidelines for implementing the EOL criteria are necessary to enhance consistency, transparency, and the accuracy of reimbursement decisions
UK [21]	A good death: non-negotiable personal conditions for clinicians, healthcare administrators, and support staff	Zaman M, Andoniou E, Wind K, Gibson J, Upshur R, Rojas G, et al.	2023	Many of the conditions for a good death that healthcare professionals care about can be achieved without advanced medical facilities or specialized expertise, allowing for new support services to make a dignified death possible for everyone, regardless of location
UK [22]	Views, Attitudes and Challenges When Supporting a Family Member in Their Decision to Travel to Switzerland to Receive Aid-In-Dying	Sperling D	2024	Participants in the study reflected on their involvement in their relatives' suicide tourism. They also discussed the challenge of revealing the deceased's plans. Family members of those involved in suicide tourism should receive professional support
France [29]	Intentions at the End of Life: Continuous Deep Sedation and France's Claeys-Leonetti law	Farrelly-Jackson S.	2024	This study examines France's 2016 law on continuous deep sedation (CDS) for terminally ill patients, focusing on the role of intention in EOL care. It argues that the law offers new insights into CDS's ethical implications and helps address broader issues of ambiguous clinical intentions
France [30]	Legalisation of euthanasia and assisted suicide: advanced cancer patient opinions - cross-sectional multicentre study	Salas S, Economos G, Hugues D, Gilbert E, Gracia D, Poulain P, et al.	2024	This study found that while most healthy French people support legalizing euthanasia, only half of palliative care patients share this view, with medical specialists largely opposed. The key factor influencing opinions was cultural, independent of other variables, and consistent with findings in other countries. It highlights the impact of personal beliefs and values on attitudes toward euthanasia and assisted suicide
France [31]	Refractory psycho-existential distress and continuous deep sedation until death in palliative care: The French perspective	Reich M, Bondenet X, Rambaud L, Ait-Kaci F, Sedda AL, Da Silva A, Villet S, Gamblin V.	2020	Before using Continuous Deep Sedation until Death, palliative care professionals should involve psycho-oncologists. Mental health experts should explore the underlying causes of distress and evaluate the intentions behind requests. Treatment options should be considered before deciding that the distress is untreatable
Italy [38]	Italian Ministry of Health. Legge sul consenso informato e sulle AHD	https://www.salute.gov.it/portale/dat/dettaglioContenutiDat.jsp?lingua=italiano&id=4953&ar ea=dat&menu=vuoto	2017	The Italian law 219/2017 allows every adult to express, through AHDs, their wishes regarding medical treatments in case of future incapacity. The AHDs are binding for healthcare providers and are accompanied by the appointment of a proxy to ensure the implementation of the patient's wishes
Italy [40]	Law n. 38. Enacted on 15th March 2010. Disposizioni per garantire l'accesso alle cure palliative e alla terapia del dolore	Available online: https://www.gazzettaufficiale.it/gunewsletter/dettaglio.jsp?service=1&datagu=2010-03-19&task=dettaglio&numgu=(10G0056) 65&redaz=010G0056&tmstp=1269600292070	2010	Law No. 38 of 2010 ensures access to palliative care and pain therapy for all patients, promoting the integration of these treatments into the national healthcare system. The law aims to improve the quality-of-life for terminally ill patients through a multidisciplinary approach and the right to dignity at the EOL
Italy [44]	Euthanasia and physician-assisted suicide for patients with depression: thought-provoking remarks	Montanari Vergallo G, Gulino M, Bersani G, Rinaldi R.	2020	Euthanasia and medical assistance in dying raise complex ethical, moral, and clinical challenges, especially when psychiatric disorders affect decision-making. The authors emphasize the importance of mental competence assessments, caution against subjective biases from doctors, and advocate for palliative care and strong social policies as alternatives to aid in dying

## 4 Discussion

The management of EOL care for cancer patients presents complex challenges that intersect with legal, ethical, and cultural considerations. The right to choose to live with dignity during the terminal phases of life, as enshrined by national and international institutions, represents a significant advancement in both medicine and the right to health. In recent decades, palliative care has emerged as an indispensable moral imperative as well as a human right within healthcare systems [16,46,47,48,49].

The alleviation of suffering represents, in all countries, a moment of “care” for the patient, aimed at achieving their well-being in a “holistic” sense, encompassing both physical and psychological aspects [5,31]. However, financial limitations and insufficient support often hinder the widespread availability of these services. The implementation of palliative care is essential to meet the growing demand and ensure access to supportive treatments for all patients facing a terminal illness that impacts their quality of life. Indeed, since health should not be considered as the mere absence of disease but as the psycho-physical well-being of the individual, both national and international human rights charters enshrine the right to live with dignity during the final stages of life [50].

All of this represents an effort toward the holistic integration of the right to health and the humanization of medicine. The right to die, and to choose to do so in the least painful manner possible for terminal patients, has become the subject of debate, much like many other rights that were once considered taboo or unthinkable [9,10,17,51,52,53,54,55,56,57]. This evolution addresses the need to ensure physical, psychological, and moral well-being for terminal patients [58,59].

This aligns with the principles of care quality and equity of access to such care. All of this goes hand in hand with the evolution of the doctor-patient relationship, which has shifted toward a system that places the individual's self-determination at its core. The journey has been long, starting with the establishment of the European Association for Palliative Care in 1988, which was the first in Europe to promote the recognition of human dignity as a cornerstone principle of the right to be free from suffering. Later, the WHO's technical report in 1990 further promoted the use of these types of care [60,61].

The increased interest and the current exponential rise in the need for palliative care may be due to both the gradual aging of the population in economically developed countries and the growing number of disabled patients suffering from chronic and oncological diseases, for which there is no effective therapy. The research conducted by the Worldwide Hospice Palliative Care Alliance estimates that in Europe, there are 560 patients per 100,000 inhabitants who require palliative care. Of these, 40% suffer from oncological diseases, thus reinforcing the concept that a significant portion of patients in need of palliative care are affected by cancer [62,63,64,65].

Studies show that the simultaneous delivery of active oncological treatment along with palliative care allows the patient to enjoy better quality of life and life expectancy, assessed through a multidimensional evaluation of needs [66,67,68]. However, despite the widespread international recognition of the importance of palliative care, there is still disparity and difficulty in accessing it in different regions of the world [69,70]. Among the factors that determine this phenomenon are, first and foremost, the lack of financial resources, and also the instability of the political situation or the presence of more urgent social problems, as can occur in countries in war situations. Probably, another contributing factor to this situation is the lack of training and education in palliative care in medical schools [71,72,73]. Our research highlights how the approach to EOL decisions in Europe differs based on national regulations, ethical frameworks, and cultural values. By focusing on Italy, France, and the United Kingdom (UK), the fundamental objective was to gain insight into how these countries navigate the delicate balance between patient autonomy, medical responsibility, and legal boundaries. In Italy, the approach to EOL care considers euthanasia and PAS illegal and focuses on palliative care. Access to these services seems uneven, due to different decisions made on a territorial basis, leading to discrepancies in interpretation of the current law. In France, the Leonetti law allows for the withdrawal of treatments and deep sedation, but euthanasia remains prohibited. The focus here is also on patient dignity and relief from suffering in cases with poor prognosis. In the United Kingdom, patient autonomy is fundamental, with a strong emphasis on advance directives and palliative care, although challenges remain in accessing services in a timely manner.

From a medico-legal perspective, the evolution of palliative care and the recognition of the right to live and die with dignity raise a debate concerning professional responsibility, the protection of patient rights and privacy, and the guarantee of equitable access to care. Decisions regarding EOL care, including palliative care, euthanasia, and PAS, must be adequately regulated to avoid conflicts between the patient's wishes, the legal responsibilities of healthcare providers, and state regulations.

In particular, the responsibility of healthcare professionals in EOL contexts is a sensitive issue, as decisions may involve the withdrawal of life-sustaining treatments, the administration of sedatives or drugs that may accelerate the dying process, and the management of potential requests for euthanasia or assisted suicide [30,44,58]. Physicians often need to strike the right balance between acting in compliance with laws that prohibit practices such as euthanasia and ensuring that patients do not suffer unnecessarily (*primum non nocere*) [74,75]. In this context, palliative care is viewed as an appropriate legal and ethical response, ensuring that terminally ill patients receive the best possible care without violating moral or legal standards. Regarding euthanasia and PAS, legislation varies from country to country, but the primary legal issues relate to the responsibility of those providing assistance and the need for fully informed and voluntary consent [76,77,78] from the patient, which should, as in all medical fields, be clear, simple, comprehensible to the patient, up-to-date, and personalized [79,80,81,82]. In countries where euthanasia is legalized, it is essential that strict criteria are followed to prevent abuse and ensure that the practice is applied only in well-documented cases that comply with current laws. Legal responsibility is, therefore, closely tied to a thorough assessment of the patient's clinical condition, verification of their will to die, and adherence to all prescribed legal procedures. In countries where euthanasia is illegal, healthcare professionals are required to balance the desire to alleviate suffering with the need to comply with legal requirements, often facing ethical dilemmas where the line between palliative care and facilitating death may appear blurred [83,84,85]. In these cases, the legal responsibility of physicians is a highly debated issue, as EOL care must be delivered in full compliance with legal norms, ensuring clear, documented informed consent, failure of which could lead to serious legal consequences for healthcare providers. Such standards are of utmost importance and increasingly relevant, as medical advances and highly innovative healthcare techniques and procedures come to deeply impact the very essence of human nature, at the end as well as beginning of life [86,87,88,89]. The growing complexity and far-reaching ramifications of such a revolution are bound to lead to major legal, ethical, and social implications, as new standards to govern it must be set to ensure its equitable and beneficial implementation for the sake of all.

## 5 Conclusion

The evolution of palliative care and the recognition of the right to live with dignity in the terminal phases of life represent a significant advancement in medicine and the right to health, reinforcing the importance of a holistic approach to patient well-being. The growing demand for these services, driven by an aging population and the rise of chronic diseases, necessitates the urgent improvement of the availability and access to palliative care, which should be considered a fundamental human right. Despite the progress made, serious inequalities in access to these treatments still persist, due to factors such as the scarcity of financial resources, political instability, and the lack of specific training in schools. The recognition and promotion of palliative care must go hand in hand with the dissemination of health policies that promote equitable and universal access to these services, through effective allocation of resources and continuous integration of professional training for all healthcare providers. In this context, ongoing international collaboration and commitment to a more humanized medicine centered on the dignity of the patient, even in some cases keeping alive and fostering hopes for a cure, are essential tools to ensure that palliative care becomes a reality for all those who need it. At the same time, the debate on euthanasia and assisted suicide has emerged as a crucial issue in modern societies, where the right to life and patient autonomy may come to clash with the ethical questions surrounding EOL assistance. Euthanasia, which involves direct medical intervention to cause the patient's death, and assisted suicide, whereby the doctor provides the patient with the means to end their own life, continue to spark spirited debate and elicit strong reactions in many countries, with legislative frameworks varying widely. In some states, euthanasia is legalized under specific circumstances, with strict legal and ethical protocols. However, in many other countries, including those we have analyzed, such practices remain illegal, and the prevailing approach is palliative care, focusing on improving the “residual” quality-of-life. While euthanasia and assisted suicide policies raise complex issues and challenges, it is important that they are considered within the context of a healthcare system that also promotes universal access to palliative care. Only a medicine that respects human dignity, offers psychological support, and upholds the rights of cancer patients can ensure a balanced and humane approach to EOL cases. This is why the evolution of health policies should include a reflection on how to adequately respond to requests for assisted death, balancing the needs of compassion, justice, and respect for patient autonomy. From a medico-legal perspective, it is essential that the healthcare system can protect both patient rights, ensuring access to appropriate care, and healthcare providers, who must act in accordance with the prevailing laws and ethical guidelines. The creation of clear regulations, which distinctly separate legal practices from illegal ones, can help prevent legal conflicts and enhance the safety and quality of EOL care.

## 6 Limitations

The selection of countries was random and geographically based. We noticed at the conclusion of our research that all three countries had a similar view on euthanasia being illegal, which contrasts with the situation in some other European countries. Nevertheless, this article focuses on a comparison between states concerning the ethical and medico-legal aspects of EOL care, which remains relevant even when considered solely from this perspective. Therefore, the authors decided not to include a country where euthanasia is legal. We intend to reserve this idea for future comparative studies by expanding our sample.
